# A Genetic Toxicology Study of the Rapid Detection of Nitrosamine Compounds by the *rpsL* Gene Mutation Assay

**DOI:** 10.3390/foods11131893

**Published:** 2022-06-26

**Authors:** Xueling Peng, Junshuai Zhang, Qingdai Liu

**Affiliations:** 1 School of Food and Biology Engineering, Xuzhou University of Technology, Xuzhou 221008, China; 2 Key Laboratory of Food Nutrition and Safety, Tianjin University of Science & Technology, Tianjin 300071, China; kaopuman@gmail.com

**Keywords:** genotoxicity test, nitrosamine, *rpsL*, safety assessment

## Abstract

In a *rpsL* gene mutation experiment, the mutagenicity of the nitrosamine compounds *N*-diethylnitrosamine (NDEA) and *N*-dipropylnitrosamine (NDPA) was investigated at the cellular level, as well as with PCR (polymerase chain reaction) and RCA (rolling-circle amplification) amplification systems. The experiments were set up with 10 ppm, 100 ppm, and 1000 ppm concentration gradients of NDEA and NDPA, and ethidium bromide (EB) was used as a positive control group. The results demonstrated that the mutagenic frequency of NDEA and NDPA was significantly higher than the spontaneous mutation frequency of the *rpsL* gene under the same conditions, but lower than the mutagenic rate of EB in the positive control, and there was a dose-effect relationship, indicating that NDEA and NDPA could induce *rpsL* gene mutation. The *rpsL* mutation system has a low spontaneous mutation background and high sensitivity, thus the system is expected to become an effective tool for the rapid detection of carcinogens in the field of food.

## 1. Introduction

Nitrosamines are a class of compounds with the structural formula R_2_ (R_1_) N-N=O. They are one of the four major food contaminants and an important class of strong chemical carcinogens [1,2]. Nitrite, nitrate, and amines, the precursors for the formation of nitrosamines, are ubiquitous in food, so the toxicology of nitrosamines has attracted extensive attention from researchers [3].

The toxicity of nitrosamines to humans and animals mainly includes acute toxicity and carcinogenicity [4]. The entry of nitrosamines into humans and animals mainly causes hepatic lobular damage and central necrosis, manifested as dizziness and fatigue, liver lesions and necrosis, pleural and ascites, and jaundice. In addition to acute toxicity, nitrosamines have broad-spectrum and strong carcinogenicity. Studies have found that preserved fish, vegetables, and smoked foods are rich in nitrite and nitrosamines [5,6], and the intake of such foods is closely related to gastric and esophageal cancer [7].

Nitrosamines need metabolic activation in the body to play a carcinogenic role. Its carcinogenicity has obvious organotropism, and long-term or excessive ingestion of nitrosamines can induce tumors in animals and humans [8]. Nitrosamine is a relatively stable compound, and its carcinogenic mechanism is as follows: the hydrogen on the carbon atom connected to ammonia nitrogen in the compound is subjected to the action of liver microsome P450, and the hydrogen on the carbon is oxidized to form a hydroxyl group, further decomposes, and undergoes isomerization into alkyl azohydroxyl compounds; it is a highly active carcinogen. It should be noted that its carcinogenicity is related to its chemical structure, physicochemical properties, and metabolic processes in vivo [4,9].

In the toxicological evaluation for food safety, the Ames genotoxicity test is often used to screen for mutagenic chemicals [10]. However, the Ames test has its inherent limitations: there are few types of mutations that can be detected, and in the Ames test, the revertant generation is random. At present, there is still a lack of research on the mutagenic mechanism of nitrosamines from the perspective of molecular biology, so it is necessary to use gene mutation experiments to study the genetic toxicology of nitrosamines.

The *rpsL* gene mutation detection system designed and used in this paper is a highly sensitive and simple genotoxicity detection method, that can provide a fast and effective way to detect potential carcinogens generated during food processing. The *rpsL* (ribosomal protein small subunit) gene encodes the S12 protein in the 30 S subunit of the ribosome. The S12 protein acts at the beginning of protein translation, and the action site of antibiotics such as streptomycin is the S12 protein in the 30 S subunit of the ribosome [11]. Under normal circumstances, the combination of streptomycin and S12 protein makes the protein biosynthesis unable to proceed, and the cell stops growing; the mutation of the *rpsL* gene makes the streptomycin loose effective binding with S12 protein, so that the strain with this gene mutation has resistance to streptomycin. It can grow on plates containing streptomycin, so mutant strains were selected [12,13]. The host strain MF101 used in this experiment has streptomycin resistance, and the plasmid pMOL21 contains ampicillin resistance and the *rpsL* gene sequence [14]. Due to the recessive genetic expression of streptomycin resistance, only when the *rpsL* gene segment of the plasmid is mutated will the host cell have streptomycin resistance, to achieve the purpose of forward screening of the mutant *rpsL* gene.

In this experiment, we selected two representative nitrosamines, NDEA and NDPA [15], as the research objects. Using the *rpsL* gene mutation detection system, through the change in resistance caused by gene mutation, the detection system of mutant strains was positively selected to realize the rapid detection of the genotoxicity of nitrosamine compounds and, according to the results, to study the genetic toxicology of nitrosamine compounds.

## 2. Materials and Methods

### 2.1. Strains and Plasmids

The strains and plasmids used in this experiment were donated by Professor Hisaji Maki of the Nara University of Science and Technology, Japan [16,17].

The mutagenicity detection of nitrosamine compounds was carried out using the *rpsL* gene on plasmid pMOL21 as a selectable marker. The ribosomal protein S12 encoded by the *rpsL* gene can form a complex with streptomycin and enhance the ability of streptomycin to bind to 16 S rRNA, thereby preventing the initiation of transcription. When the *rpsL* gene is mutated, the interaction between 16 S rRNA and streptomycin is disrupted, resulting in cell resistance to streptomycin.

We adopted *Escherichia coli* MF101, whose *rpsL* gene is mutated, so this strain has streptomycin resistance. The plasmid pMOL21 contains ampicillin as a selection marker and the correct *rpsL* gene. When the pMOL21 plasmid is transformed into the host cell, due to the recessive genetic expression of streptomycin, only when the *rpsL* gene on the plasmid is mutated will the host cell have streptomycin resistance, to achieve the purpose of forward screening of the mutant *rpsL* gene. For specific information on strains and plasmids, please refer to [14].

### 2.2. Determination of the Mutation Rate of the rpsL Gene at the Cellular Level

*E. coli* MF101 + pMOL21 were cultured with different concentrations (10 ppm, 100 ppm, or 1000 ppm) of NDEA or NDPA. Ten hours after growth, the strains were collected and spread on plates containing ampicillin (Amp^+^, 50 μg/mL) to determine the total number of transformed cells and spread on plates containing ampicillin and streptomycin (Amp^+^, Str^+^, 50 μg/mL) to determine the number of colonies with *rpsL* gene mutations. The mutation rate is the ratio of the number of mutant colonies to the total number of colonies. Each group of experiments was repeated five times, and the total number of colonies was not less than 10^6^. The experimental data and images were statistically analyzed by Origin2018 software.

### 2.3. Determination of the rpsL Gene Mutation Rate in the PCR Amplification System

The PCR system was as follows: 10 × PCR buffer (Mg^2+^) 5 μL, dNTP 2 μL, pMOL21 plasmid template 1 μL, upstream and downstream primers 1 μL, *Pfu* DNA polymerase 0.5 μL, ddH_2_O supplemented to 20 μL (nitrosamine compounds were directly added to the PCR system).

The PCR program was as follows: predenaturation at 95 °C for 5 min, denaturation at 94 °C for 30 s, annealing at 55 °C for 30 s, extension at 72 °C for 120 s, 25 cycles, full extension at 72 °C for 10 min, and storage at 16 °C.

**Figure 1:**
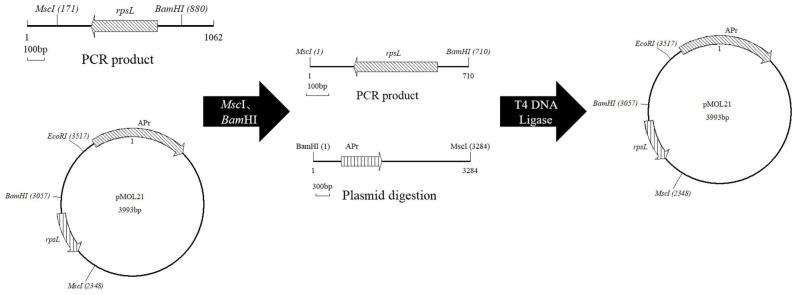
Flow chart of the treatment of PCR products.

The PCR products were recovered and ligated with restriction enzymes (*Msc*I, *Bam*H1), transformed into MF101 strains, plated on ampicillin-containing plates to determine the total number of transformed cells, and plated on ampicillin- and streptomycin-containing plates to identify *rpsL* gene-mutated colonies. A flow chart of the treatment of PCR products is provided in Figure 1.

### 2.4. Determination of the rpsL Gene Mutation Rate in the RCA Reaction System

Heat denaturation of samples and annealing reaction system of primers and plasmids: 1 μL of 10 × phi29 DNA polymerase reaction buffer, 2.5 μL of 100 μM random primers, 1 μL of plasmid pMOL21 to be amplified, and 4.3 μL of ddH_2_O. The above system was heated at 95 °C for 3 min and then placed on ice for 15 min.

Amplification reaction system: 10 mM dNTP 0.5 μL, 100 × BSA 0.2 μL, phi29 DNA polymerase 0.5 μL to the above reaction solution (nitrosamine compounds or EB are added to the amplification reaction system), and then incubated at 30 °C overnight.

Heat-inactivated phi29 DNA polymerase: heat at 65 °C for 10 min.

**Figure 2:**
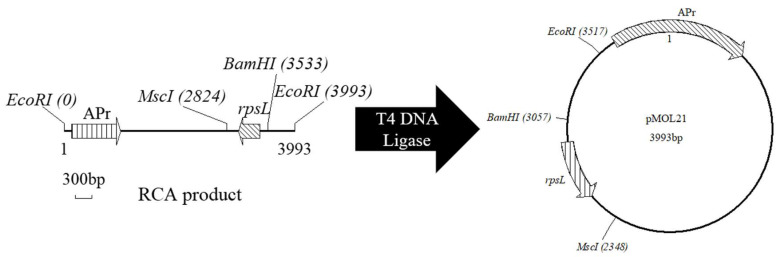
Flow chart of the treatment of RCA products.

A flow chart of the treatment of RCA products is provided in Figure 2.

## 3. Results and Discussion

### 3.1. Determination of the rpsL Gene Replication Spontaneous Mutation Rate in E. coli MF101

*E. coli* was grown without a mutagen and screened by Amp^+^ and Amp^+^&Str^+^ resistance plates, and the colonies were counted. The spontaneous mutation rate of the *rpsL* gene was 0.70 × 10^−6^. The above experiments were repeated four times, and the average number of strains in each group of parallel controls reached 10^8^. The data are provided in Table 1.

### 3.2. Statistics of Spontaneous Mutation Types of rpsL Gene Duplication

Through large-scale sequencing, we determined the *rpsL* gene sequence in a total of 500 mutant strains, compared them with WT, and obtained the type of spontaneous mutation in the process of *rpsL* gene replication. The statistical results are found in Table 2.

**Figure 3:**
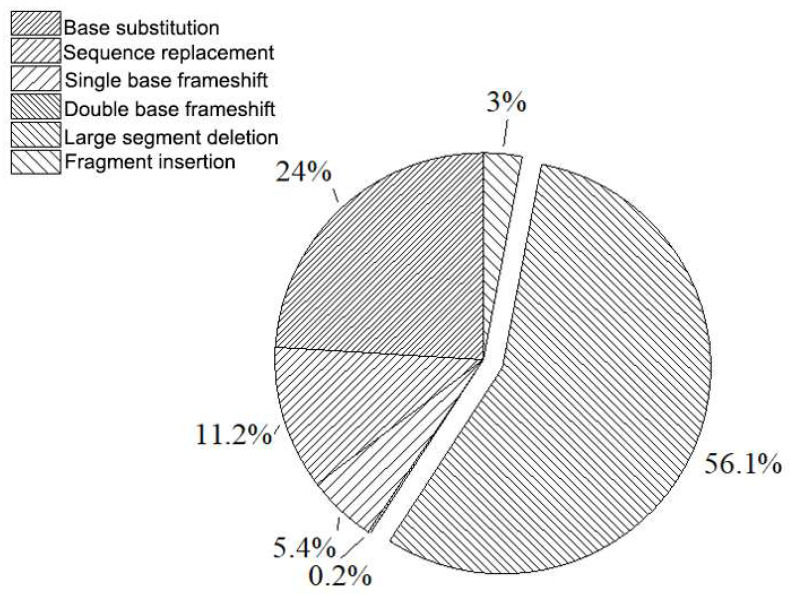
Statistics of spontaneous mutation types of *rpsL*.

Studies have demonstrated that, during the replication process of the *rpsL* of *E. coli*, the proportion of spontaneous mutation types of large fragment deletion is the highest, reaching 56%, followed by base substitution, sequence substitution, frameshift mutation, and large fragment deletion, the four main types of gene mutation, as well as insertion mutation (the data are provided in Figure 3). Among them, gene mutation types that Ames cannot detect (large fragment deletion, sequence substitution, fragment insertion, etc.) accounted for more than 70%. This indicates that the *rpsL* detection system avoids the limitation of mutation types in the Ames test and is suitable for the analysis and detection of trace mutagenic substances.

### 3.3. Detection of the Induced Mutation Rate of rpsL Gene Replication at the Cellular Level

EB was used as a mutagenic positive control, and the *rpsL* mutation system was used to detect the mutation rate of *rpsL* induced by it at the cellular level. The experimental data obtained are provided in Table 3. At concentrations of 10 ppm, 100 ppm, and 1000 ppm EB, the mutation rates of the *rpsL* gene in *E. coli* were 3.71 ± 0.502 × 10^−6^, 5.78 ± 0.728 × 10^−6^, and 7.87 ± 1.842 × 10^−6^, respectively. The above results demonstrated that EB had strong mutagenicity at lower concentrations, and this mutagenicity increased with increasing concentration.

**Figure 4:**
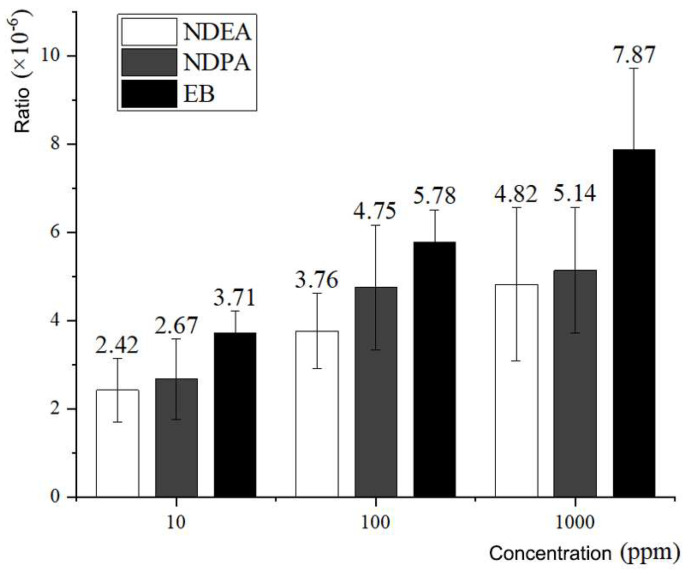
Comparison of *rpsL* mutation rates induced by NDEA, NDPA and EB.

The results of the *rpsL* gene mutation induced by nitrosamine compounds at the cellular level demonstrated that NDEA and NDPA had high genotoxicity at a concentration of 10 ppm, and the induced *rpsL* gene mutation rates were 2.42 ± 0.717 × 10^−6^ and 2.67 ± 0.909 × 10^−6^, respectively, which were significantly higher than the spontaneous mutation rate of *rpsL* under the same conditions (0.70 ± 0.322 × 10^−6^). The mutation rates of the *rpsL* gene induced by NDEA and NDPA at 100 ppm and 1000 ppm were 3.76 ± 0.852 × 10^−6^ and 4.75 ± 1.417 × 10^−6^, 4.82 ± 1.738 × 10^−6^ and 5.14 ± 1.433 × 10^−6^, respectively. This result illustrates that the genotoxicity of NDEA and NDPA to *E. coli* DNA replication increases with increasing concentration. The higher the concentrations of NDEA and NDPA were, the stronger the mutagenicity, and the difference in the mutation rate between the two was greater. These results indicate that the mutagenicity of NDPA at the cellular level is higher than that of NDEA. The detailed experimental data are provided in Table 3; Figure 4 illustrates the statistics of the data in Table 3.

### 3.4. Detection of the Spontaneous Mutation Rate of rpsL Gene Replication in the PCR System

Compared with *Taq* DNA polymerase, *Pfu* DNA polymerase has excellent thermal stability and proofreading properties, and its amplification error rate is stable at approximately 10^−6^ [18]. The *rpsL* detection system in this experiment uses *Pfu* DNA polymerase to amplify the *rpsL* gene in the PCR system. The results demonstrated that the spontaneous mutation rate of the *rpsL* gene in the PCR system was 1.30 ± 0.635 × 10^−6^ (the detailed experimental data are provided in Table 4), which was basically consistent with the previously reported *Pfu* DNA polymerase amplification error rate.

### 3.5. Detection of the Induced Mutation Rate of rpsL Gene Replication in the PCR System

**Figure 5:**
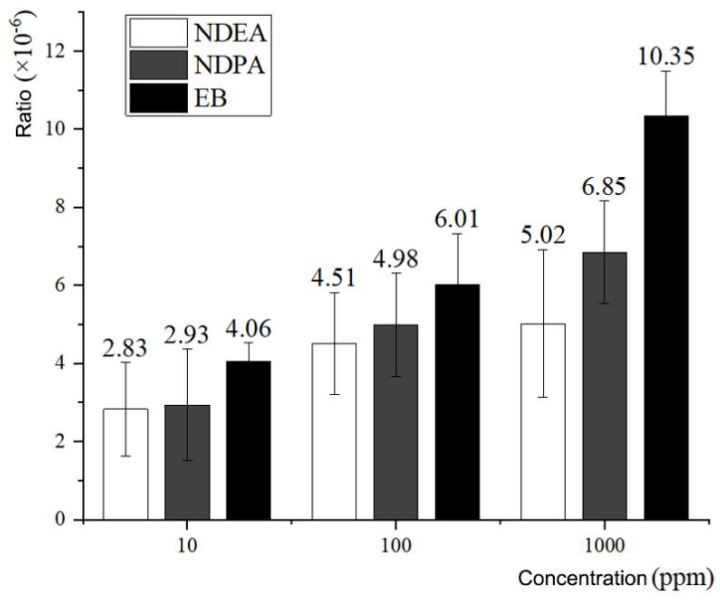
Comparison of *rpsL* mutation rates induced by NDEA, NDPA, and EB by PCR.

We measured the mutation rate of mutagen-induced *rpsL* gene mutations in the PCR system. The mutagen was added directly to the PCR system to determine the mutation rate of the *rpsL* gene during DNA amplification. The results are as follows: the mutation rates of *rpsL* induced by NDEA and NDPA at concentrations of 10 ppm, 100 ppm, and 1000 ppm were 2.83 ± 1.197 × 10^−6^ and 2.93 ± 1.430 × 10^−6^, 4.51 ± 1.314 × 10^−6^ and 4.98 ± 1.330 × 10^−6^, and 5.02 ± 1.890 × 10^−6^ and 6.85 ± 1.316 × 10^−6^, respectively. The mutation rates of the *rpsL* gene induced by positive control EB with the same concentration gradient were 4.06 ± 0.471 × 10^−6^, 6.01 ± 1.319× 10^−6^, and 10.35 ± 1.143 × 10^−6^, respectively (the detailed experimental data are provided in Table 5; Figure 5 illustrates the statistics of the data in Table 5). In the PCR system, the mutagenic rate of NDEA was lower than that of NDPA, but it was lower than that of EB in the positive control group.

### 3.6. Determination of the Spontaneous Mutation Rate of rpsL in the RCA System

Unlike traditional DNA amplification techniques, such as the PCR system, RCA is an isothermal nucleic acid amplification technique in which the polymerase continuously adds nucleotides to a circular template, resulting in a long tandem repeat (ssDNA) containing tens to hundreds of tandem repeats (complementary to the circular template) [19]. The DNA polymerases used in RCA are phi29, Bst, and Vent (exo-), but since phi29 DNA polymerase has the best processivity and strand displacement ability among the above polymerases, it is commonly used in the RCA reaction process [20,21,22]. Unlike PCR systems, RCA can be performed at a constant temperature (from room temperature to 37 °C) [23]. In addition, RCA can amplify a single molecule binding event a thousand-fold, making it suitable for the detection of ultralow abundance targets [24]. Currently, RCA, as a general signal amplification tool, has a wide range of applications in genomics, proteomics, diagnostics, and biosensing [25,26].

We used the RCA system to determine the spontaneous mutation rate of the *rpsL* gene, and the result, provided in Table 6, was 1.09 ± 0.456 × 10^−6^. The amplification mutation rate of the *rpsL* gene under the action of *Pfu* DNA polymerase was 1.30 ± 0.635 × 10^−6^, which was one order of magnitude lower than that of *Taq* DNA polymerase, which was 10^−5^.

### 3.7. Detection of the Induced Mutation Rate of the rpsL Gene in the RCA System

We then determined the mutation rate of mutagen-induced *rpsL* gene mutations in the RCA system. In this experiment, we adopted the method of directly adding a mutagen to the RCA amplification reaction solution to provide a direct mutagenic environment for the amplification of the *rpsL* gene. This method avoids the possibility of affecting the mutagenicity of NDEA, NDPA, or EB during thermal denaturation.

**Figure 6:**
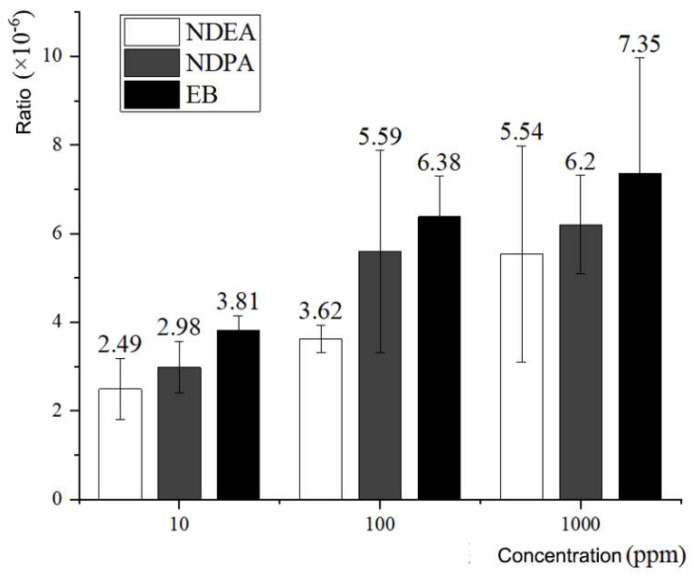
Comparison of *rpsL* mutation rates induced by NDEA, NDPA, and EB in the RCA reaction.

The experimental results were as follows: the *rpsL* gene mutation rates induced by NDEA and NDPA at concentrations of 10 ppm, 100 ppm, and 1000 ppm were 2.49 ± 0.692 × 10^−6^ and 2.98 ± 0.585 × 10^−6^, 3.62 ± 0.316×10^−6^ and 5.59 ± 2.287 × 10^−6^, and 5.54 ± 2.444 × 10^−6^ and 6.20 ± 1.110 × 10^−6^, respectively. The mutation rates of the *rpsL* gene induced by the positive control EB with the same concentration gradient were 3.81 ± 0.335 × 10^−6^, 6.38 ± 0.915 × 10^−6^, and 7.35 ± 2.615 × 10^−6^, respectively (the detailed experimental data are provided in Table 7; Figure 6 illustrates the statistics of the data in Table 7). In the RCA process, the mutagenicity of NDEA was lower than that of NDPA, and both were lower than that of EB in the positive control group.

## 4. Conclusions

We determined the type of mutation in the *rpsL* gene in *E. coli.* From the sequencing results, it can be observed that there are many types of spontaneous mutations in the process of *rpsL* gene replication in *E. coli*, but the Ames test cannot detect nearly 70% of the gene mutations, and the *rpsL* detection system avoids the limitations of this detection method. This result demonstrates that the *rpsL* detection system is suitable for the analysis and detection of microbial mutagens.

We determined that the spontaneous mutation rate of the *rpsL* gene in *E. coli* was 0.70 × 10^−6^, while the mutation rate of the *rpsL* gene induced by EB was 3.71 ± 0.502 × 10^−6^; the mutagenicity results demonstrated that the mutation rate of the *rpsL* gene reached 2.42 ± 0.717 × 10^−6^ and 2.67 ± 0.909 × 10^−6^ when 10 ppm NDEA and NDPA were added, respectively.

In the PCR system, the amplification error rate of *Pfu* DNA polymerase was stable at approximately 10^−6^ [27], while the spontaneous mutation rate of the *rpsL* gene in the PCR system we determined was 1.30 ± 0.635 × 10^−6^, which is consistent with a previous report. The amplification error rate of *Pfu* DNA polymerase is basically the same. When 10 ppm EB was added to the PCR system, the mutation rate of the *rpsL* gene was 4.06 ± 0.471 × 10^−6^; when 10 ppm nitrosamine compound NDEA or NDPA was added, the mutation rate of the *rpsL* gene was 2.83 ± 1.197 × 10^−6^ and 2.93 ± 1.430 × 10^−6^, respectively.

The spontaneous mutation rate of the *rpsL* gene in the RCA reaction system was 1.09 ± 0.456 × 10^−6^, which was lower than the amplification mutation rate of the *rpsL* gene in the PCR system (1.30 ± 0.635 × 10^−6^) and one order of magnitude lower than the mutation rate of the *rpsL* gene by *Taq* DNA polymerase [27]. In the RCA system, when EB was added at a concentration of 10 ppm, the mutation rate of the *rpsL* gene reached 3.81 ± 0.335 × 10^−6^; when 10 ppm NDEA and NDPA were added, the mutation rate of the *rpsL* gene reached 2.49 ± 0.692 × 10^−6^ and 2.98 ± 0.585 × 10^−6^, respectively.

The above results illustrated that the mutagenic rate of NDEA and NDPA increased with increasing concentration, indicating that nitrosamines could induce mutation of the *rpsL* gene. At the same time, it was found that the mutagenicity of nitrosamine at a concentration of 10 ppm was still significantly higher than the spontaneous mutagenicity of the *rpsL* gene, but it was still lower than the mutagenicity of EB, indicating that the system has the characteristics of a low spontaneous mutation background and high sensitivity.

Previous studies have proposed that the determination of NDPA and NDEA in drinking water and beer can be performed using solid-phase micro-extraction (SPME) along with gas chromatography (GC) and mass spectrometry (MS), and the process can be completed within 70 min [28]. However, the disadvantage is that the requirements for instruments and equipment are relatively high, and some laboratories may not have this equipment. In addition, studies have proposed the use of host-mediated assays (HMAs) for the toxicological analysis of this compound [29]. However, this method involves mouse experiments. First, the experimental period is long. In addition, it also considers the limitations of mouse feeding conditions in some laboratories. There are also experiments using the classical Ames test to measure the mutagenicity of five *N*-nitrosamines (*N*-nitrosodimethylamine (NDMA), NDEA, NDPA, *N*-nitrosopyrrolidine (NPYR), and *N*-nitrosodiphenylamine (NDPhA)), but their results indicate that the formation of mutagens during UV photolysis was detected only in the case of NDPhA in strain TA98. The oxidation products of NDMA, NDEA, and NDPhA did not indicate any significant mutagenicity in the strains used, whereas the oxidation of NDPA and NPYR by hydroxyl radicals seems to lead to the formation of direct mutagens in YG7108 and TAMix [30].

Previous studies have applied *rpsL* gene mutation to the detection and analysis of chromosomal DNA mutations [31] and to detect the mutagenicity of compounds in the aquatic environment [32]. However, there is no relevant report on the quantitative determination of the mutagenicity of compounds using this system. Commonly, mutagenicity evaluation systems mainly use mammalian cell forward mutation experiments, namely hypoxanthine guanine phosphoribosyl transferase (HPRT) gene and thymidine kinase (TK) gene mutation experiments [33]. However, these experiments all require complex mammalian culture conditions, and the experimental process is mostly more than 10 days. The *rpsL* system was used for detection, and the whole analysis process was realized within 24 h. At the same time, the spontaneous mutation of the *rpsL* is low. For example, the spontaneous mutation rate of the TK gene is 10^−5^, while the spontaneous mutation rate of *rpsL* is as low as 10^−6^, which is much lower than that of similar screening systems. In addition, using the *rpsL* positive selection system to evaluate the mutagenicity caused by nitrosamine compounds does not require complicated experimental conditions, saving manpower and material resources. Therefore, the *rpsL* gene mutation assay is expected to become an effective tool for the rapid detection of carcinogens in the food industry.

## Figures and Tables

**Table 1:** Spontaneous mutation rate of *rpsL*.

Total Strain (×10^6^)	Mutant Strain	Ratio (×10^−6^)	
121	98	0.81	0.70 ± 0.322
257	153	0.60	
94	102	1.09	
205	67	0.33	

**Table 2:** Statistics of spontaneous mutation types of *rpsL*.

Types	Mutant Strains	Ratio (%)
Base substitution	120	24
Sequence replacement	56	11.2
Single base frameshift	27	5.4
Double base frameshift	1	0.2
Large segment deletion	280	56.1
Fragment insertion	15	3

**Table 3:** Mutation rate of *rpsL* induced by mutagens.

Concentration	Mutagen	Mutant Strains	Total Strains (10^5^)	Mutation Rate (10^−6^)	
10 ppm	NDEA	120	832	1.44	2.42 ± 0.717
		129	552	2.34	
		104	352	2.95	
		40	135	2.96	
	NDPA	125	735	1.70	2.67 ± 0.909
		182	650	2.80	
		265	687	3.86	
		200	856	2.34	
	EB	307	840	3.65	3.71 ± 0.502
		310	708	4.38	
		224	616	3.64	
		245	775	3.16	
100 ppm	NDEA	250	752	3.32	3.76 ± 0.852
		159	566	2.81	
		208	492	4.23	
		304	648	4.69	
	NDPA	332	580	5.72	4.75 ± 1.417
		213	800	2.66	
		344	676	5.09	
		420	760	5.53	
	EB	330	508	6.50	5.78 ± 0.728
		184	304	6.05	
		260	544	4.78	
		272	470	5.79	
1000 ppm	NDEA	140	484	2.89	4.82 ± 1.738
		140	364	3.85	
		272	452	6.02	
		280	428	6.54	
	NDPA	289	410	7.05	5.14 ± 1.433
		136	324	4.20	
		160	296	5.41	
		218	560	3.89	
	EB	110	112	9.82	7.87 ± 1.842
		51	76	6.71	
		76	128	5.94	
		109	121	9.01	

**Table 4:** Spontaneous mutation rate of *rpsL* in PCR.

Total Strains (10^6^)	Mutant Strains (×10)	Mutant Rate (10^−6^)	
87	70	0.80	1.30 ± 0.635
27	41	1.52	
23	18	0.78	
19	40	2.11	

**Table 5:** Mutation rate of *rpsL* induced by mutagens in PCR.

Concentration	Mutagen	Mutant Strains	Total Strains (10^5^)	Mutation Rate (10^−6^)	
10 ppm	NDEA	90	705	1.28	2.83 ± 1.197
		163	614	2.65	
		173	421	4.11	
		60	182	3.30	
	NDPA	148	769	1.92	2.93 ± 1.430
		204	668	3.05	
		340	693	4.91	
		175	954	1.83	
	EB	356	841	4.23	4.06 ± 0.471
		346	774	4.47	
		275	662	4.15	
		273	807	3.38	
100 ppm	NDEA	315	851	3.70	4.51 ± 1.314
		185	599	3.09	
		272	497	5.47	
		403	699	5.77	
	NDPA	387	628	6.16	4.98 ± 1.330
		258	839	3.08	
		391	717	5.45	
		427	819	5.21	
	EB	363	606	5.99	6.01 ± 1.319
		273	355	7.69	
		281	629	4.47	
		334	568	5.88	
1000 ppm	NDEA	168	531	3.16	5.02 ± 1.890
		156	424	3.68	
		343	551	6.23	
		307	437	7.03	
	NDPA	321	416	7.72	6.85 ± 1.316
		342	421	8.12	
		191	302	6.32	
		310	590	5.25	
	EB	206	172	11.98	10.35 ± 1.143
		64	176	9.32	
		137	135	10.15	
		169	170	9.94	

**Table 6:** Spontaneous mutation rate of *rpsL* in the RCA reaction.

Total Strains (10^6^)	Mutant Strains	Mutation Rate (10^−6^)	
113	74	1.53	1.09 ± 0.456
61	45	1.36	
42	84	0.50	
60	62	0.97	

**Table 7:** Mutation rate of *rpsL* induced by mutagens in the RCA reaction.

Concentration	Mutagen	Mutant Strains	Total Strains (10^5^)	Mutation Rate (10^−6^)	
10 ppm	NDEA	158	881	1.79	2.49 ± 0.692
		174	863	2.02	
		191	597	3.20	
		212	716	2.96	
	NDPA	264	735	3.59	2.98 ± 0.585
		251	823	3.05	
		305	985	3.10	
		187	856	2.18	
	EB	317	876	3.62	3.81 ± 0.335
		343	796	4.31	
		242	669	3.62	
		303	821	3.69	
100 ppm	NDEA	261	753	3.47	3.62 ± 0.316
		231	683	3.38	
		210	592	3.55	
		240	588	4.08	
	NDPA	354	722	4.90	5.59 ± 2.287
		268	697	3.85	
		489	546	8.96	
		351	751	4.67	
	EB	393	541	7.26	6.38 ± 0.915
		263	395	6.66	
		283	555	5.10	
		310	478	6.49	
1000 ppm	NDEA	132	421	3.14	5.54 ± 2.444
		141	356	3.96	
		191	290	6.59	
		270	319	8.46	
	NDPA	301	387	7.78	6.20 ± 1.110
		187	361	5.18	
		242	406	5.96	
		257	438	5.87	
	EB	127	185	6.86	7.35 ± 2.615
		59	145	4.07	
		152	147	10.34	
		159	196	8.11	

## Data Availability

Data is contained within the article.
